# Acute Retinal Necrosis: Signs, Treatment, Complications and Outcome

**DOI:** 10.3390/diagnostics12020386

**Published:** 2022-02-02

**Authors:** Christian S. Mayer, Katharina Blobner, Julia Storr, Isabella D. Baur, Ramin Khoramnia

**Affiliations:** 1 University Eye Clinic Heidelberg, Heidelberg University, Im Neuenheimer Feld 400, 69120 Heidelberg, Germany; Christian.Mayer@med.uni-heidelberg.de (C.S.M.); Isabella.Baur@med.uni-heidelberg.de (I.D.B.); 2 Department of Ophthalmology, Klinikum Rechts der Isar München der Technischen Universität München, Ismaninger Straße 22, 81675 Munich, Germany; katharina.blobner@icloud.com (K.B.); Julia.Storr@mri.tum.de (J.S.)

**Keywords:** acute retinal necrosis, uveitis, Varicella-Zoster-Virus

## Abstract

Background: The Acute Retinal Necrosis (ARN) is an inflammatory, rapidly progressive necrotizing retinitis and vasculitis, most frequently caused by Varicella-Zoster-Virus (VZV), followed by Herpes-Simplex-Virus (HSV), Cytomegalovirus (CMV) and Epstein-Barr-Virus (EBV). The diagnosis is based on clinical signs that were first defined by the American Uveitis Society in 1994 that include one or more foci of retinal necrosis, rapid progression without treatment, circumferential progression, occlusive vasculopathy, and inflammatory signs of the vitreous and anterior chamber Methods: In this retrospective analysis, we included 16 eyes of 10 patients, six patients with simultaneous or delayed bilateral affection, treated for ARN. Status of disease, corrected distance visual acuity (CDVA, decimal), intraocular pressure (IOP), pathogen proof, therapy, and complications were evaluated at diagnosis and 3 months later. Results: In nine patients, the pathogen was identified (six VZV, two HSV, one CMV, one EBV). All patients were treated with systemic and intravitreal virustatic agents. In nine eyes with a CDVA of 0.2 ± 0.2 at hospital admission, vitrectomy was performed, and in seven eyes with CDVA of 0.5 ± 0.3, no vitrectomy was performed (*p* = 0.04). After 3 months, CDVA of the vitrectomized eyes decreased to 0.1 ± 0.1 vs. 0.4 ± 0.3 (*p* = 0.01) without vitrectomy. CDVA of fellow eyes affected was 0.6 ± 0.2 at initial presentation vs. 0.2 ± 0.2 for eyes affected first and 0.4 ± 0.3 vs. 0.1 ± 0.1 after 3 months. We observed several complications including retinal detachment, recurrence of the disease, and bulbar hypotony. Conclusion: For fellows eyes affected, diagnosis could be confirmed earlier, leading to a more successful treatment. The success of vitrectomy is difficult to evaluate because vitrectomy is most frequently performed just in the advanced stages of the disease. Early treatment with an appropriate approach is essential to avoid loss of vision.

## 1. Introduction

Acute Retinal Necrosis (ARN) is a rare and rapidly progressive, inflammatory, vision-threatening disease with low incidence [1] in otherwise healthy people. The American Uveitis Society established a widely accepted definition of the disease based solely on clinical findings [2]. ARN is usually triggered by the α herpes viruses varicella zoster virus (VZV) and herpes simplex virus type 1 or type 2 (HSV-1, HSV-2). Cytomegalalovirus (CMV), which is one of the β herpes viruses, plays a marginal role in the development of ARN. The role of the Epstein-Barr virus (EBV), which is associated with the γ herpes viruses, in the development of ARN is controversially discussed [3,4]. Combined infections (e.g., VZV and CMV or EBV) have been reported [5]. Patients of all ages and sexes can be affected. It is mainly described in immunocompetent patients, but exceptions are also known [6,7]. The disease begins unilaterally with pain and anterior uveitis. Usually there is a strong vitreous reaction and peripheral areas of whitish retinal infiltration, which confluence and spread to the posterior pole. Typically, there is an occlusive vasculitis, and more rarely an opticopathy [8]. Because of the aggressive progression of this destructive disease, an early diagnosis specific treatment as early as possible are necessary to preserve the best possible visual acuity due to remission of the inflammation [9]. Therapy includes parenteral antiviral therapy in the acute phase, followed by maintenance therapy to prevent recurrence and infestation of the fellow eye. As it is well known that the fellow eyes can also be affected in up to 75% of patients [10,11,12], an early antiviral treatment can have a protective effect on the fellow eye [13]. In addition, correct and early therapy may reduce the risk for complications such as retinal detachment [14], which occurs in up to 75% of all cases and presents a major cause of poor visual outcomes [15]. Peripheral retinal tears can be treated with Argon laser retinopexy under antiviral therapy. Vitrectomy with silicone oil tamponade can result in retinal reattachment in over 90% of patients, but the prognosis in terms of corrected distance visual acuity (CDVA) outcome is very limited.

Although a number of case studies and review articles on ARN have been published in recent years, there are still no internationally accepted diagnostic and therapeutic guidelines. This is primarily due to the rarity of the disease. Two national studies in the United Kingdom found an incidence of 0.5 to 0.63 cases per million [5,16]. Most knowledge about ARN comes from small case series [14,17]; data from controlled trials are lacking and therefore many questions remain unanswered. Diagnostic problems can occur, especially in the early stages, and differential diagnosis can be difficult.

As ARN presents a rare, but serious disease, we reviewed all cases of ARN treated at a German university eye clinic between 2009 and 2019. We evaluated disease status at the initial diagnosis and at the 3 months follow-up. We investigated whether vitrectomy was performed and whether this had an impact on CDVA outcomes.

## 2. Methods

In this case series, we retrospectively reviewed patients with diagnosed and documented clinical signs of ARN over a period of 10 years (January 2009 to December 2019). Twelve patients were identified, of whom 10 patients could be included in this analysis. Two patients were excluded: one patient died after the first ophthalmologic visit in course of an exacerbating acute myeloid leukemia and the other one denied treatment and did not show up for any follow-up visit. Characteristic markers were vision loss in one or even both eyes, moderate pain, any kind of anterior chamber or vitreous inflammation, vasculitis, and retinitis with multilocal, mainly peripheral beginning and progressive retinal infiltrations. Diagnosis was based on the criteria defined by the American Uveitis Society: at least one focus of retinal necrosis, rapid progression without therapy, circumferential spread of the disease, occlusive arteriolar vasculopathy, and inflammatory reaction in the vitreous and anterior chamber [2]. Every diagnosis was supported by imaging, optical coherence tomography (OCT), and fluorescein angiography (FA).

Assessed parameters included demographics, CDVA (decimal) at first presentation and after 3 months, diagnostic findings like macular involvement, pathogen proof, therapy, and common complications during treatment like abnormalities in terms of intraocular pressure (IOP) or retinal detachment. We examined if any surgical treatment was necessary. Especially intravitreal injection of antiviral medication or even more invasive procedures such as vitrectomy were considered to be of particular interest.

Therapy was based on the recommendations in the literature [9,18]. Initial drug treatment included weight adapted (10 mg/kg body weight) parenteral Aciclovir and intravitreal Ganciclovir injections. Later, the therapy was switched to oral administration and continued for several weeks or months depending on clinical findings. During hospitalization, clinical course was observed by daily examination of anterior chamber and fundi with dilated pupils.

For sample collection from the infiltrated vitreous, either a one-port anterior vitrectomy or even a full vitrectomy was performed. The sample was sent to the department of virology in an insulin syringe for analysis using polymerase chain reaction (PCR). Sampling, injection, and vitrectomy were performed in the operating room under sterile conditions.

Data are presented as means ± standard deviation. The main outcome variable was CDVA. Tested treatment variables were antiviral intravitreal injections alone vs. antiviral intravitreal injections after vitrectomy. We also compared the outcomes of the eye affected first to those of the fellow eye that was affected second. Visual acuity, which was too low to be assessed with visual acuity charts, was qualitatively graded as counting fingers, hand motion, light perception-only, and no light perception discriminated. These informatively missing visual acuities were imputed as graded worst ranks [19]. Statistics were performed using SPSS version 26.0 for MAC (IBM Corp., Armonk, NY, USA).

## 3. Results

**Figure 1:**
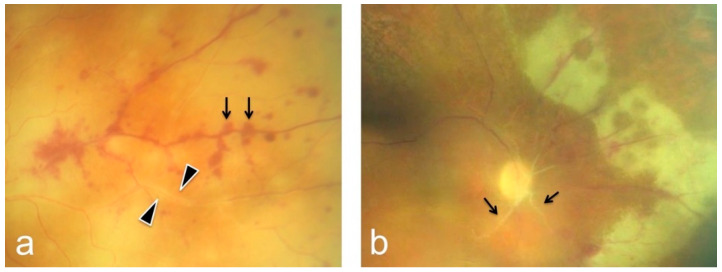
Findings in acute retinal necrosis with retinitis and perivascular infiltrations (arrowheads) as well as perivascular hemorrhage, whitish fundus, and retinal edema (**a**). (**b**) Parapapillary occlusive vasculitis, pale optic nerve disc, and peripheral retinal inflammation with secondary necrosis.

**Figure 2:**
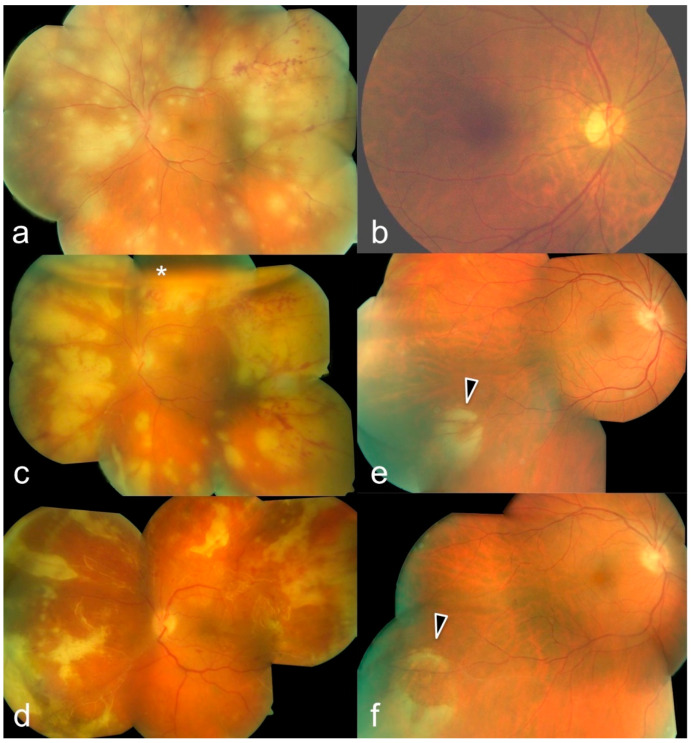
59-year-old patient with acute retinal necrosis (ARN) in the left eye: first presentation with suspected retinal branch artery occlusion and iritis treated with topical prednisolone for about 10 days and “non improving vision”. (**a**) Fundus photography of the left eye. Visual acuity 0.5; conjunctiva injected, anterior chamber cells +, Arlt’s triangle with pattern of mutton-fat keratic precipitates, retina with circular whitish foci and hemorrhages, especially localized temporally. (**b**) The right eye was not affected. Treatment including vitrectomy, intravitreal ganciclovir, and air (asterisk) (**c**) followed by detection of varizella zoster virus (VZV) using polymerase chain reaction (PCR) and parenteral aciclovir treatment (10 mg/kg of body weight every 8 h + prednisolone and two ganciclovir intravitreal injections. Ten days later, retinal detachment occurred and was treated with vitrectomy, ganciclovir injection, and silicone oil tamponade. The retina was stable postoperatively, and the visual acuity was 0.2 (**d**). Four weeks after the onset of the ARN in the left eye, the right eye achieved a visual acuity of 1.0, but showed a temporal fresh necrotic focus (**e**, arrowhead). Immediate start with intravitreal Ganciclovir. Follow-up 10 days later with a clearly demarcated infiltrate (**f**) followed by weekly checkups and long-term treatment with 800 mg Aciclovir five times a day. For further details see Table 1, Patient 4.

**Figure 3:**
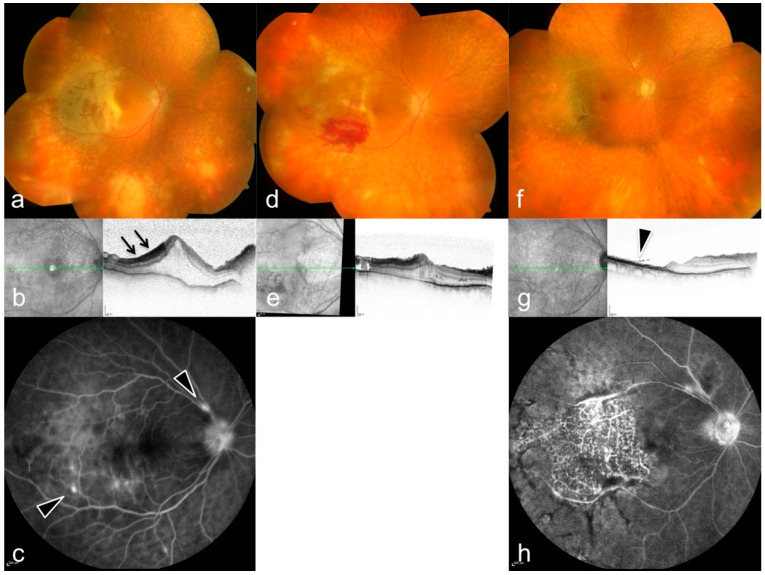
Bilateral onset of acute retinal necrosis (ARN). Right eye findings on first presentation: (**a**) Funduscopy with peripheral retinal infiltrations, (**b**) ischemic inner retinal layers (arrows) and edema in optical coherence tomography (OCT), (**c**) vascular leakage in fluorescein angiography (arrowheads). (**d**–**h**) follow-up with resolving infiltrates, development of bleedings (**d**), and retinal necrosis (**g**), arrowhead. For further details see Table 1, patient 8.

**Figure 4:**
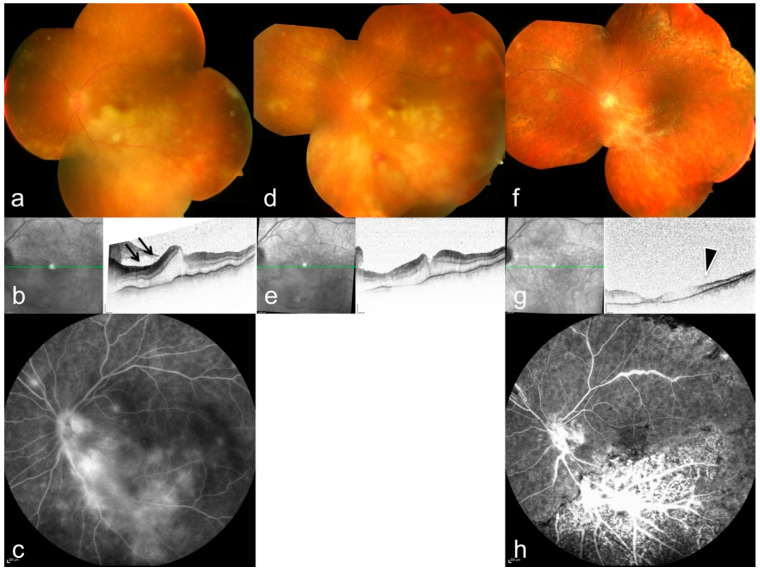
Corresponding left eye of the patient in Figure 3 on first presentation: (**a**) funduscopy with peripheral retinal infiltrations, (**b**) ischemic inner retinal layers (arrows), and edema in optical coherence tomography (OCT), (**c**) vascular leakage in fluorescein angiography (arrowheads). (**d**–**h**) follow-up with resolving infiltrates, development of bleedings (**d**), and retinal necrosis (**g**), arrowhead. For further details see Table 1, patient 8.

A total of 10 patients (five males, five females) with clinical signs of an ARN could be included (Figure 1). Six patients (60%) showed bilateral affection (Figure 2, Figure 3 and Figure 4). Therefore, a total of 16 eyes could be assessed. Table 1 summarizes the obtained results. In patients with a bilateral disease, a simultaneous onset was detected in three patients. In three patients, a latency of 1, 2, and 31 months was noted, respectively. All eyes presented with anterior chamber cells, vitreous cells, and retinal signs such as bleeding, vasculitis, and retinal necrosis developing from the periphery to the center. In three eyes, a moderately elevated intraocular pressure (IOP) was found at time of diagnosis.

In nine patients, the pathogen was identified (six VZV, two HSV, one CMV, one EBV) using PCR. In one patient with bilateral affection no pathogen could be identified despite vitreous aspiration.

All patients were treated with systemic and intravitreal virustatic agents. Parenteral Aciclovir at a dosage of 10 mg/kg of body weight was administered for at least 5 days three times a day. Depending on the clinical signs therapy was switched to oral administration. Intravitreal injection with Ganciclovir was performed 3.5 mm away from the limbus with adosage of 2 mg/0.5 mL. If necessary, a paracentesis was created.

Because of the severity of the disease, vitrectomy was performed in nine eyes with pronounced vitritis or retinal detachment. In case of a vitrectomy a gas tamponade (C2F6) was primarily used (after reattachment of the retina in cases with retinal detachment) to facilitate treatment with intravitreal injections in the course of the disease. Only in case of complicated, old, or recurrent retinal detachment we decided intraoperatively to use a silicone oil filling (Siluron 5000).The Supplementary Video (Video S1) shows intraoperative findings during vitrectomy in a patient with severe ARN. CDVA at hospital admission was 0.2 ± 0.2 in these eyes. In the remaining seven eyes, CDVA was better (0.5 ± 0.3; *p* = 0.04) and no vitrectomy was performed. These eyes were treated with at least one intravitreal injection of Ganciclovir. Visual acuity of the six fellow eyes at hospital admission was 0.6 ± 0.2. A vitrectomy was only performed in two of these six eyes.

After 3 months, the CDVA of the vitrectomized eyes had decreased to 0.1 ± 0.1, while the CDVA in the eyes without vitrectomy was 0.4 ± 0.3 (*p* = 0.01).

An affection of the fellow eye was always detected at an earlier stage of the disease compared to the initial manifestation. CDVA at hospital admission was 0.6 ± 0.2 vs. 0.2 ± 0.2 in cases, where the fellow eye was not affected. In these bilateral cases, final CDVA was 0.1 ± 0.1 in the first affected eye and 0.4 ± 0.3 in the fellow eye with a later onset of the disease.

Several complications during the course of treatment were observed. One eye developed a relevant cataract and another eye an IOL dislocation. After 3 months, two vitrectomized eyes were hypotonic (IOP < 10 mmHg). In total, 6 of 16 eyes had a retinal detachment. Retinal detachment occurred in two of nine eyes with, and in four of seven eyes without previous vitrectomy. Only one eye presented with a documented recurrence after 3 months.

## 4. Discussion

ARN is an acute viral retinitis and an ophthalmologic emergency requiring immediate diagnosis and treatment. In 1971, Urayama et al. described six patients with acute unilateral retinitis, retinal arteritis, choroiditis, and later onset of retinal detachment [20]. 11 years later, Culbertson et al. were able to attribute the etiology of this rare, until then completely unclear, disastrous disease to an infectious genesis by electron microscopic detection of varicella zoster viruses (VZV) in all retinal layers [21]. The disease and progression of ARN is frequently associated with a relevant loss of vision. The often poor functional outcome is probably primarily a consequence of the usually extensive ischemia of the retina, choroid, and especially the optic nerve in severe cases [14,17].

Six patients (60%) showed bilateral affection, although the infection started predominantly unilaterally. However, in the further course, the partner eye was affected in up to 30–75% [1,10,11,12,13]. This was confirmed by our data. The interval to second eye disease can be as long as 30 years [13,22]. In six cases in our cohort, the second eye was affected, but with less severe symptoms and a milder course. Lei et al. described this phenomenon before: [10] whenever the partner eye is affected, the diagnosis can be confirmed earlier. Thus, a faster and more successful treatment can be achieved. This is also one of the reasons why the success of vitrectomy cannot be fully evaluated. Vitrectomy is more often performed when the disease has progressed and BCVA is already severely reduced.

The diagnosis of ARN can often be made clinically. ARN should be distinguished from progressive outer retinal necrosis (PORN), which was first described in 1990 [23,24] and occurs in immunocompromised patients (e.g., caused by HIV). Differential diagnoses are also CMV retinits, Toxoplasmosis, M. Behçet, and sarcoidosis. Although diagnosis of ARN is based on clinical signs, we recommend pathogen detection in order to ascertain the diagnosis and initiate a targeted antiviral therapy. Specific antibody analysis and detection of the virus genome by PCR in the aqueous humor and/or vitreous have proven to be diagnostic tools with high sensitivity and specificity [1]. In contrast, serological tests are of little help in detecting an active viral infection. Diagnostic samples can initially be obtained by anterior chamber tap using paracentesis, by vitreous fine needle aspiration or, in advanced stages, with therapeutic pars plana vitrectomy. The significantly improved possibilities of intraocular virus detection lead to a detection rate of up to 80–90% [1,25,26,27]. In our cohort, the pathogen was identified in nine patients (six VZV, two HSV, one CMV, one EBV) using PCR. From a therapeutic point of view, a differentiation between HSV-1, HSV-2, and VZV is of less importance, because the antiviral therapy does not differ in these two pathogens. In contrast, virological and clinical differentiation of CMV retinitis from ARN caused by the three α-herpesviruses is important, because ganciclovir is the drug of choice in CMV infections [4].

Once ARN is diagnosed, antiviral therapy should be started immediately to prevent disease progression and prophylactic long-term therapy should be considered, ideally using valaciclovir due to its better bioavailability. In our cohort, we used the antiviral drugs recommended in literature [9,18]. However, the ideal antiviral therapeutic regime for treatment of ARN remains unclear. All our patients were initially treated with intravenous aciclovir followed by oral therapy. Newer antiviral agents such as valaciclovir or famciclovir have a higher bioavailability when administered orally; therefore, new treatment algorithms using solely oral virustatic agents have been adopted in the last decades [5,28,29]. This approach permits to avoid hospital admission. Randomized controlled clinical trials examining the effectiveness are still lacking, however. Permanent virustatic therapy as well as the use of systemic and topic corticosteroids and systemic antiplatelet agents is controversially discussed [5,14,30,31].

Recurrent retinal detachment is a frequent complication of ARN. Kopplin et al. found that 75% of patients suffer from retinal detachment in the course of the disease requiring surgery [32]. Dave et al. [33] found a rate of recurrent retinal detachment of 18.75% up to 59.09% depending on the severity of the disease at initial presentation [33]. In our study, only one patient had recurrent retinal detachment in one eye (6.25% of eyes). This difference might be related to the smaller sample size in our study. Lau et al. reported the possibility of prophylactic laser retinopexy to prevent retinal detachment [14]. Despite surgical intervention with pars-plana-vitrectomy, visual results can be limited in those cases.

In summary, despite all advances in drug and surgical therapy, ARN remains a serious and often life-altering disease. Parenteral antiviral therapy supplemented by intravitreal injections are of utmost therapeutic importance. The fastest possible diagnosis and immediate initiation of therapy are crucial. Special attention should be paid to sufficient antiviral drug levels during the first weeks of the disease. Furthermore, life-long therapy to protect the fellow eye is controversially discussed. Vitreoretinal surgery can reattach the retina in a high percentage but cannot restore the visual loss caused by ARN.

## Figures and Tables

**Table 1:** Data of 10 patients and 16 eyes with ARN. Vitrectomy (vitr.), intravitreal injection (IVT), herpes simplex virus (HSV), varizella-zoster-virus (VZV), Eppstein-Barr-virus (EBV), best corrected visual acuity (BCVA, Snellen chart), intraocular lens (IOL), therapy (Tx).

#	Sex	Age	Eye	BCVA at Onset	Local Tx	Systemic Tx	Pathogen	Complications	Timepoint of 2nd Involvement	BCVA after 3 Months
1	M	53	OD	0.6	vitrectomy	Aciclovir	HSV	none	3 months later	0.2
			OS	0.2	vitrectomy			IOL luxation	-	0.1
2	M	54	OD	0.5	IVT Ganciclovir	Aciclovir	EBV, VZV	none	7 weeks later	0.8
			OS	0.1	vitrectomy + IVT			cataract surgery	-	0.2
3	M	65	OS	0.063	IVT Ganciclovir	Aciclovir	HSV	multiple retinal detachments	-	hand motion
4	M	59	OD	1.0	IVT Ganciclovir	Aciclovir	VZV	none	1 month later	1.0
			OS	0.6	vitrectomy + IVT Ganciclovir			retinal detachment	-	0.2
5	M	66	OD	0.3	IVT Ganciclovir	Aciclovir	VZV	retinal detachment	-	0.2
6	F	86	OD	0.4	vitrectomy + IVT Ganciclovir	Aciclovir	-	Vitreous hemorrage, vitr. Silicone oil tamponade, endophthalmitis, secondary glaucoma, retinal branch occlusion	same time	0.4
			OS	0.1	vitrectomy + IVT Ganciclovir			Vitreous hemorrage, vitr. Silicone oil tamponade	-	0.5
7	F	76	OD	0.5	IVT Ganciclovir	Aciclovir	CMV	none	same time	light perception
			OS	0.1	vitrectomy + IVT Ganciclovir			Recurrence 3 months later	-	light perception
8	F	77	OD	0.4	IVT Aciclovir	Aciclovir	VZV	none	same time	0.5
			OS	hand motion	vitrectomy + IVT Ganciclovir			retinal detachment	-	counting fingers
9	F	60	OS	hand motion	vitrectomy + IVT Ganciclovir	Aciclovir	VZV	retinal detachment	-	light perception
10	F	32	OD	0.6	IVT Ganciclovir	Aciclovir	VZV	retinal detachment	-	counting fingers

## Data Availability

The data presented in this study are available on request from the corresponding author. The data are not publicly available due to data protection regulations.

## Supplementary Materials

The following supporting information can be downloaded at: https://www.mdpi.com/article/10.3390/diagnostics12020386/s1. Video S1: Intraoperative findings during vitrectomy in a patient with severe ARN.

## References

[1] Gandorfer A., Thurau S. (2009). Acute retinal necrosis. Ophthalmology.

[2] Holland G.N. (1994). Standard Diagnostic Criteria for the Acute Retinal Necrosis Syndrome. Am. J. Ophthalmol.

[3] Bonfioli A.A., Eller A.W. (2005). Acute retinal necrosis. Semin. Ophthalmol..

[4] Rautenberg P., Grancicova L., Hillenkamp J., Nölle B., Roider J., Fickenscher H. (2009). Acute retinal necrosis from the virologist’s perspective. Ophthalmol. Z. Dtsch. Ophthalmol. Ges..

[5] Cochrane T.F., Silvestri G., McDowell C., Foot B.E., McAvoy C. (2012). Acute retinal necrosis in the United Kingdom: Results of a prospective surveillance study. Eye.

[6] Freeman W.R., Lerner C.W., Mines J.A., Lash R.S., Nadel A.J., Starr M.B., Tapper M.L. (1984). A Prospective Study of the Ophthalmologic Findings in the Acquired Immune Deficiency Syndrome. Am. J. Ophthalmol..

[7] Freeman W.R., O’Connor G.R. (1984). Acquired Immune Deficiency Syndrome Retinopathy, Pneumocystis, and Cotton-Wool Spots. Am. J. Ophthalmol..

[8] Pleyer U., Metzner S., Hofmann J. (2009). Diagnostics and differential diagnosis of acute retinal necrosis. Ophthalmol. Z. Dtsch. Ophthalmol. Ges..

[9] Hillenkamp J. (2009). Diagnosis and therapy of acute retinal necrosis. Ophthalmol. Z. Dtsch. Ophthal-Mologischen Ges..

[10] Lei B., Jiang R., Wang Z., Xu G., Wu X., Zhou M. (2020). Bilateral Acute Retinal Necrosis: A Case Series. Retina.

[11] Williams A.M., Nguyen V.Q., Botsford B.W., Eller A.W. (2020). Bilateral acute retinal necrosis caused by two separate viral etiologies. Am. J. Ophthalmol. Case Rep..

[12] Young N.J., Bird A.C. (1978). Bilateral acute retinal necrosis. Br. J. Ophthalmol..

[13] Palay D.A., Sternberg P., Davis J., Lewis H., Holland G.N., Mieler W.F., Jabs D.A., Drews C. (1991). Decrease in the Risk of Bilateral Acute Retinal Necrosis by Acyclovir Therapy. Am. J. Ophthalmol..

[14] Lau C.H., Missotten T., Salzmann J., Lightman S.L. (2007). Acute Retinal Necrosis: Features, Management, and Outcomes. Ophthalmology.

[15] Butler N.J., Moradi A., Salek S.S., Burkholder B.M., Leung T.G., Dunn J.P., Thorne J.E. (2017). Acute Retinal Necrosis: Presenting Characteristics and Clinical Outcomes in a Cohort of Polymerase Chain Reaction–Positive Patients. Am. J. Ophthalmol..

[16] Muthiah M.N., Michaelides M., Child C.S., Mitchell S.M. (2007). Acute retinal necrosis: A national population-based study to assess the incidence, methods of diagnosis, treatment strategies and outcomes in the UK. Br. J. Ophthalmol..

[17] Hillenkamp J., Nölle B., Bruns C., Rautenberg P., Fickenscher H., Roider J. (2009). Acute Retinal Necrosis: Clinical Features, Early Vitrectomy, and Outcomes. Ophthalmology.

[18] Yeh S., Suhler E.B., Smith J.R., Bruce B., Fahle G., Bailey S.T., Hwang T.S., Stout J.T., Lauer A.K., Wilson D.J. (2014). Combination systemic and intravitreal antiviral therapy in the management of acute retinal necrosis syndrome. Ophthalmic Surg. Lasers Imaging Retin..

[19] Lachin J.M. (2020). Worst-Rank Score Methods—A Nonparametric Approach to Informatively Missing Data. J. Am. Med. Assoc..

[20] Urayama A. (1971). Unilateral acute uveitis with retinal periarteritis and detachment. Rinsho Ganka (Jpn. J. Clin. Ophthalmol.).

[21] Culbertson W.W., Blumenkranz M.S., Haines H., Gass J.D.M., Mitchell K.B., Norton E.W. (1982). The acute retinal necrosis syndrome: Part 2: Histopathology and etiology. Ophthalmology.

[22] Falcone P.M., Brockhurst R.J. (1993). Delayed onset of bilateral acute retinal necrosis syndrome: A 34-year interval. Ann. Ophthalmol..

[23] Forster D.J., Dugel P.U., Frangieh G.T., Liggett P.E., Rao N.A. (1990). Rapidly progressive outer retinal necrosis in the acquired immunodeficiency syndrome. Am. J. Ophthalmol..

[24] Matsuo T., Morimoto K., Matsuo N. (1991). Factors associated with poor visual outcome in acute retinal necrosis. Br. J. Ophthalmol..

[25] Harper T.W., Miller D., Schiffman J.C., Davis J.L. (2009). Polymerase Chain Reaction Analysis of Aqueous and Vitreous Specimens in the Diagnosis of Posterior Segment Infectious Uveitis. Am. J. Ophthalmol..

[26] Müller B., Velhagen K., Pleyer U. (2000). Acute retinal necrosis syndrome: Analysis, therapy and long-term follow up of 14 eyes. Klin. Mon. Augenheilkd..

[27] Gargiulo F., De Francesco M.A., Nascimbeni G., Turano R., Perandin F., Gandolfo E., Manca N. (2003). Polymerase chain reaction as a rapid diagnostic tool for therapy of acute retinal necrosis syndrome. J. Med. Virol..

[28] Aizman A., Johnson M.W., Elner S.G. (2007). Treatment of Acute Retinal Necrosis Syndrome with Oral Antiviral Medications. Ophthalmology.

[29] Emerson G.G., Smith J.R., Wilson D.J., Rosenbaum J.T., Flaxel C.J. (2006). Primary treatment of acute retinal necrosis with oral antiviral therapy. Ophthalmology.

[30] Tibbetts M.D., Shah C.P., Young L., Duker J.S., Maguire J.I., Morley M.G. (2010). Treatment of Acute Retinal Necrosis. Ophthalmology.

[31] Kawaguchi T., Spencer D.B., Mochizuki M. (2008). Therapy for acute retinal necrosis. Seminars in Ophthalmology.

[32] Kopplin L.J., Thomas A.S., Cramer S., Kim Y.H., Yeh S., Lauer A.K., Flaxel C.J. (2016). Long-Term Surgical Outcomes of Retinal Detachment Associated With Acute Retinal Necrosis. Ophthalmic Surg. Lasers Imaging Retin..

[33] Dave V.P., Pappuru R.R., Pathengay A., Tyagi M., Narayanan R., Jalali S. (2019). Vitrectomy with Silicone Oil Tamponade in Rhegmatogenous Retinal Detachment following Acute Retinal Necrosis: Clinical Outcomes and Prognostic Factors. Seminars in Ophthalmology.

